# Proteomic profiling of the weed feverfew, a neglected pollen allergen source

**DOI:** 10.1038/s41598-017-06213-z

**Published:** 2017-07-20

**Authors:** Isabel Pablos, Stephanie Eichhorn, Peter Briza, Claudia Asam, Ulrike Gartner, Martin Wolf, Christof Ebner, Barbara Bohle, Naveen Arora, Stefan Vieths, Fatima Ferreira, Gabriele Gadermaier

**Affiliations:** 1 University of Salzburg, Department of Molecular Biology, Division of Allergy and Immunology, Salzburg, Austria; 2 University of Salzburg, Department of Ecology and Evolution, Salzburg, Austria; 3 Allergy Clinic Reumannplatz, Vienna, Austria; 4 Department of Pathophysiology and Allergy Research, Medical University of Vienna, Vienna, Austria; 5 CSIR-Institute of Genomic and Integrative Biology, Allergy and Immunology Section, Delhi, India; 6 Paul-Ehrlich-Institut, Federal Institute for Vaccines and Biomedicines, Langen, Germany

## Abstract

Feverfew *(Parthenium hysterophorus)*, an invasive weed from the Asteraceae family, has been reported as allergen source. Despite its relevance, knowledge of allergens is restricted to a partial sequence of a hydroxyproline-rich glycoprotein. We aimed to obtain the entire sequence for recombinant production and characterize feverfew pollen using proteomics and immunological assays. Par h 1, a defensin-proline fusion allergen was obtained by cDNA cloning and recombinantly produced in *E. coli*. Using two complementary proteomic strategies, a total of 258 proteins were identified in feverfew pollen among those 47 proteins belonging to allergenic families. Feverfew sensitized patients’ sera from India revealed IgE reactivity with a pectate lyase, PR-1 protein and thioredoxin in immonoblot. In ELISA, recombinant Par h 1 was recognized by 60 and 40% of Austrian and Indian sera, respectively. Inhibition assays demonstrated the presence of IgE cross-reactive Par h 1, pectate lyase, lipid-transfer protein, profilin and polcalcin in feverfew pollen. This study reveals significant data on the allergenic composition of feverfew pollen and makes recombinant Par h 1 available for cross-reactivity studies. Feverfew might become a global player in weed pollen allergy and inclusion of standardized extracts in routine allergy diagnosis is suggested in exposed populations.

## Introduction

*Parthenium hysterophorus* is an herbaceous weed from the Asteraceae family. It is recognized with different common names like feverfew, congress grass, the “Scourge of India”, or bitter weed. Feverfew grows in subtropical climates and originates from southern United States, Central and South America. It was accidentally introduced to Australia, Africa and India where it has become an invasive species^1^. Notably, transient populations of feverfew have recently also been described in parts of Europe, e. g. Poland and Belgium^2^. The weed can grow especially in warm climates all over the year while the typical flowering period is from July to September. Besides its beneficial use as medicinal herb, feverfew was also described to elicit allergic dermatitis due to sesquiterpene lactones^1, 3, 4^. In countries where the weed is invasive, allergic rhinitis to feverfew has been documented^3^. In a study involving 810 rhinitis patients from Bangalore, southern India, 38% were skin prick test positive to feverfew^5^, while 100 pollen allergic patients from Cuba showed a sensitization prevalence of 79% to feverfew^6^. Since 1995, allergies to feverfew pollen have been a major health problem in Queensland, Australia^7^. A more recent study revealed a sensitization prevalence of 36% among Indian patients suffering from *Parthenium* sensitive atopic dermatitis^8^. A particular drawback regarding allergy diagnosis of feverfew is the limitation of standardized and commercially available extracts and the lack of inclusion in routine diagnosis.

Additionally, there are observations suggesting a high risk of allergic reaction due to IgE cross-reactivity with other botanical related weeds, e.g. feverfew sensitized patients who were never exposed to ragweed showed positive skin prick tests to ragweed extract^9^. Moreover, IgE antibodies from ragweed allergic patients were inhibited up to 94% by feverfew pollen extract and up to 82% in a reverse experimental setup^10^. Though at present we lack epidemiologic studies on sensitization prevalence, we should be aware that the allergenicity of feverfew is likely to increase on a global basis due to climatic changes and the tremendous invasive capacity of this weed^2^.

Despite the obvious importance of feverfew in airborne allergy, little information is available on allergenic components triggering type I reactions. In 1995, Gupta *et al*. demonstrated the presence of 5 IgE reactive proteins from 14 to 45 kDa in pollen extracts of feverfew by immunoblot^11^. Later, the same group purified a hydroxyproline-rich glycoprotein from feverfew extract and revealed a partial amino acid sequence of this major allergen^12^. Though IgE binding with pooled patients’ sera was demonstrated in immunoblot, no further studies on this or any other allergen in this source were performed.

In this work, we performed a comprehensive proteomic study for identification of potentially allergenic proteins from feverfew pollen. In addition, we identified the full-length cDNA sequence of the previously described hydroxyproline-rich glycoprotein and produced a recombinant molecule. IgE immunoblot analysis and ELISA experiments were performed to investigate antibody binding and cross-reactivity with relevant allergenic molecules from mugwort and ragweed pollen.

## Methods

Detailed description of all experimental procedures is provided in the online Supplementary material and methods. All methods were performed in accordance with the relevant guidelines and regulations.

### Patients’ sera

Weed pollen-allergic individuals from Austria (n = 15) were selected on the basis of case history, i.e. recurrent rhinitis/conjunctivitis during late summer, and allergen-specific IgE to mugwort and ragweed pollen determined by ImmunoCAP (Supplementary Table S1). For the inhibition experiment, a pool of six patients’ sera was used covering sensitization to relevant mugwort and ragweed allergens (Supplementary Table S2). In addition, we screened forty-four Indian sera from allergic patients for IgE reactivity to feverfew pollen extract. Information on patients with positive reactivity (n = 25) is shown in the Table S1. Experiments using anonymized serum samples from allergic patients were approved by the ethics committee of the Medical University of Vienna, Austria (712/2010) and the CSIR-Institute of Genomics and Integrative Biology, New Delhi, India (CLP 0019). Informed written consents were obtained from all subjects.

### Pollen extracts

Feverfew *(Parthenium hysterophorus)* pollen was collected in Delhi, India. Quality and purity of pollen grains was evaluated after collection in India and Austria. For protein extraction, 1 g of feverfew pollen was shaken overnight in extraction buffer and subsequently dialyzed against 5 mM sodium phosphate pH 7.0. Pollen extracts from mugwort *(Artemisia vulgaris)* and ragweed *(Ambrosia artemisiifolia)* were prepared as previously described^13^.

### cDNA cloning of Par h 1

Pollen grains from feverfew were ground and total RNA purification was performed using TRIzol. Reverse transcription was followed by cDNA amplification with nested PCR using degenerated primers based on the previously described partial Par h 1 amino acid sequence^12^. Amplified products were cloned into the pGEM-T Easy vector and the 5′-UTR and the signal peptide sequence were retrieved using a 5′ RLM-RACE protocol. Finally, the full-length sequence was obtained using a forward gene specific primer localized in the signal peptide and reverse oligo dT.

### Production of recombinant allergens

Par h 1 mature protein sequence was cloned into pHisParallel2 vector using *Nde* I and *Xho* I as restriction enzymes. Recombinant Par h 1 was expressed as non-fusion protein in *E. coli* Rosetta-gamiB (DE3) pLysS. Soluble protein supernatants were precipitated with ammonium sulfate and Par h 1 enriched supernatant was subjected to hydrophobic interaction and size exclusion chromatography. Purified recombinant allergens from mugwort (Art v 3, Art v 4 and Art v 5) and a natural allergen from ragweed pollen (Amb a 1) were obtained as previously published^14–17^. Production of non-tagged Art v 1 is described in the online methods section.

### Physico-chemical characterization of purified Par h 1

Amino acid analysis was performed using the Pico Tag method as described^14^. For intact mass measurements, samples were desalted and directly infused into a Q-Exactive mass spectrometer. Raw data were processed with Protein Deconvolution 2.0. Circular dichroism spectra were recorded with a JASCO J-815 spectropolarimeter at 20 °C, 95 °C and after cooling down to 20 °C.

### 1D and 2D-gel electrophoresis

Feverfew pollen extract, bacterial lysates and purified Par h 1 were analyzed by 1D reducing gel electrophoresis. Proteins were visualized with Coomassie Brilliant Blue R-250 staining. For 2D gel electrophoresis, feverfew pollen grains were ground to fine powder in liquid nitrogen and pollen extract was obtained as described^18^. The vacuum dried pellet was re-suspended in isoelectric focusing buffer and proteins were loaded onto ReadyStrip IPG strip pH 3–10 using buffer compositions and isoelectric focusing conditions as previously described^13^. Afterwards, the strip was loaded onto 15% acrylamide gels, and proteins were separated by reducing SDS-PAGE. Proteins were visualized with Coomassie Brilliant Blue R-250 and protein spots were excised.

### Mass spectrometry of feverfew proteins

For proteomic analyses of feverfew pollen, two complementary methods were used. The first approach relied on generating a tryptic digest of the pollen extract which was directly analyzed by reverse-phase liquid chromatography mass spectrometry (LC-MS/MS). In this case, the protein extract from 30 mg of pollen was prepared using an extraction method previously described^18^. The other method consisted in separation of the pollen proteins by two-dimensional electrophoresis (2-DE), as described above, followed by in-gel digestion of the protein spots and mass analysis (2-DE-LC-MS/MS). Both preparations were reduced, alkylated and digested with ProteoExtract All-in-One Trypsin Digestion Kit.

### Database search and gene ontology annotations

Fragment spectra were searched against NCBInr database using PEAKS Studio 7.5 with a false discovery rate (FDR) of 1%. Identification of allergenic proteins was based on similarity search against the Allergome database (uniprot release 29.03.2016) and subsequently constraint using database entries listed at the International Union of the Immunological Societies (IUIS) allergen nomenclature sub-committee. The final list of proteins was annotated to Gene Ontology using Blast2GO.

### Immunoblot, ELISA and cross-inhibition experiments

Feverfew pollen extract and purified Par h 1 were separated in 1D gel electrophoresis, electroblotted onto nitrocellulose membrane and tested with sera of Austrian and Indian patients. Analogous, extract and purified proteins were immobilized on ELISA plates and IgE reactivity was analyzed with patients’ sera using a colorimetric or chemiluminescence detection system. For selection of sera reactive to mugwort and ragweed pollen allergens, IgE reactivity to purified recombinant Art v 1, Art v 3, Art v 4, Art v 5 and Amb a 1 was verified (Supplementary Table S2). This serum pool (n = 6) was used to perform the inhibition ELISA, testing the inhibitory capacity of pollen extract towards immobilized purified mugwort and ragweed allergens. Sera were pre-incubated with increasing concentrations (0.2–200 µg/mL) of pollen extracts from feverfew, mugwort or ragweed as well as buffer only. The inhibition ELSA was validated using unrelated inhibitor proteins, i.e. BSA and fetal calf serum (FCS).

## Results

### The full-length cDNA sequence of Par h 1 was successfully identified

**Figure 1:**
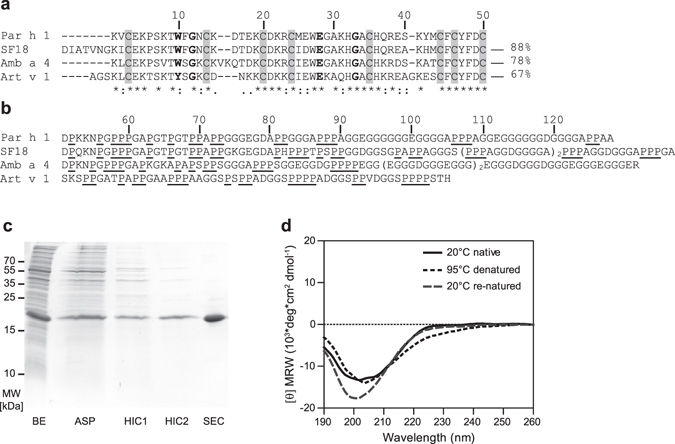
Sequence analysis, purification and secondary structure elements of recombinant Par h 1. (**a**) Sequence alignment of defensin-like domains obtained with the multiple sequence alignment program Clustal Omega (**b**), comparison of proline-rich regions. Art v 1 from *Artemisia vulgaris* (Q84ZX5), Amb a 4 from *Ambrosia artemisiifolia* (D4IHC0) and SF18 from *Helianthus annuus* (P22357) were included. Conserved cysteine residues boxed in grey, conserved residues of defensin-like proteins in bold, proline clusters underlined. The symbols shown in the alignment correspond with identical amino acids (*), conserved substitutions (:), and semi-conserved substitutions (.). (**c**) Purification process of recombinant Par h 1: BE, bacterial extract; ASP, ammonium sulfate precipitation; HIC1 and HIC2, hydrophobic interaction chromatography and SEC, size exclusion chromatography. (**d**) Circular dichroism spectra of purified Par h 1. The unprocessed gel is shown in the Related Manuscript File.

The full-length cDNA sequence of Par h 1 was obtained from total feverfew pollen RNA by nested PCR with degenerated primers followed by 5′RLM-RACE protocols. Three identical cDNA clones (GenBank KM267639) were retrieved, with an open reading frame of 468 nucleotides, encoding a protein of 156 amino acids and a predicted 28 amino acids signal peptide. The mature protein sequence consisted of 128 amino acids with a theoretical molecular mass of 12.2 kDa and pI 5.33. Par h 1 contained a defensin-like domain (residues 1–50) fused to a proline-rich region (residues 51–128) (Fig. 1a,b). Par h 1 presented conserved features of plant defensin-like proteins, i.e. eight cysteines, an aromatic amino acid at position 10, a glycine at positions 12 and 33, and an aspartic acid at position 28 (Fig. 1a)^19^. The defensin-like domain showed 67–88% sequence identity with homologous pollen proteins (Fig. 1a). The C-terminal region consisted of proline-rich repetitions, though it differed in length from other defensin-proline fusion proteins (Fig. 1b). The novel allergen was officially acknowledged by the IUIS allergen nomenclature sub-committee and is listed as Par h 1.0101.

### Recombinant Par h 1 shows a typical defensin-like fold

The sequence corresponding to mature Par h 1 was cloned into the pHisParallel 2 expression vector. For increased protein stability, a glycine residue was introduced at the N-terminus. Par h 1 was expressed as non-fusion protein in *E. coli* yielding 6.3 mg/L expression culture. Purity >98% was achieved using a purification strategy based on the combination of ammonium sulfate precipitation followed by hydrophobic interaction and size exclusion chromatography. Gel-electrophoresis showed a single protein band migrating around 20 kDa (Fig. 1c). The exact protein concentration and amino acid composition were determined by amino acid analysis. Protein identity was confirmed by intact mass spectrometry where the theoretical mass of 12,199.32 Da fitted perfectly with the experimental mass (12,199.32 Da). Circular dichroism analysis of purified Par h 1 showed a spectrum with a minimum at 202 nm which is in agreement with the typical CD spectral behavior of defensin-proline fusion proteins^20^. Upon thermal denaturation, the spectrum of Par h 1 changed in shape at the region 220–230 nm and the minimum slightly shifted to 204 nm. Upon cooling, these changes were nearly restored and only minor differences were observed (Fig. 1d).

### Proteomics of feverfew pollen reveals the presence of 47 allergenic protein families

**Figure 2:**
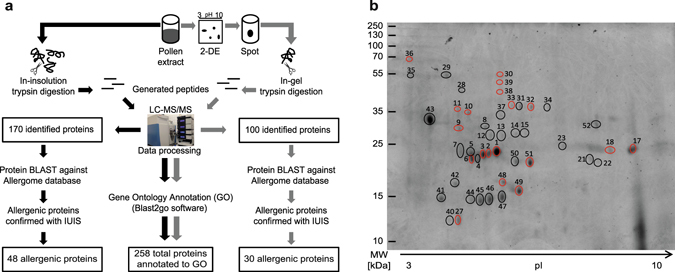
Proteomics of feverfew pollen reveals the presence of allergenic protein families. (**a**) Summarized workflow and results of the proteomic analyses. (**b**) Two-dimensional gel electrophoresis of feverfew pollen extract. After the isoelectric focusing, proteins were separated by SDS-PAGE and the spots were visualized by Coomassie staining. All excised spots subjected to mass analysis are circled and spots containing allergenic molecules are indicated in red circles. Detailed information of identified allergenic protein families is shown in Table 1. The unprocessed gel is shown in the Related Manuscript File.

Aiming to confirm the presence of Par h 1 at the protein level and investigate the presence of other putative allergenic molecules in the pollen of feverfew, we performed a proteomic study. The quality of feverfew pollen was first verified by microscopy at the collection site (India) and subsequently prior to mass analysis in Austria. Feverfew pollen showed a purity of ~99.6%, and minor contaminations with other pollen of less than 0.1% were found consisting of grains from birch, oak, ash, grass and fern, respectively (Supplementary Fig. S1). We employed two complementary proteomic approaches, LC-MS/MS and 2-DE-LC-MS/MS; workflow and overview on results are summarized in Fig. 2a. Tryptic digestion of the total extract followed by LC-MS/MS allowed identification of 170 individual protein species (Supplementary Table S3). Upon 2-DE separation, 55 protein spots were excised and further analyzed by mass spectrometry (Fig. 2a). Out of the 55 analyzed spots, 46 were considered for protein identification according to the selected cutoff ≥3 unique peptides (Fig. 2b). The Supplementary Table S3 shows all identified proteins within each spot, while Table 1 shows the spot number where the corresponding protein had the best identification score. In total, 100 non-redundant proteins were identified when combining 2-DE with LC-MS/MS (Fig. 2a and Supplementary Table S3). The presence of Par h 1 in feverfew pollen was confirmed by both strategies, where 100% sequence coverage was obtained for the defensin-like domain. Furthermore, identified proteins were compared with sequences from the Allergome database using a sequence identity cutoff ≥50%. For further consideration, the protein family had to be officially recognized by the IUIS allergen nomenclature sub-committee (Fig. 2a). The combination of the two proteomic approaches revealed 47 allergenic protein families (Table 1). Twenty-three allergenic protein families were detected with both methods. Digestion of pollen extract followed by LC-MS/MS allowed the identification of 19 allergenic protein families, while 5 were exclusively identified by 2-DE-LC-MS/MS (Table 1 and Fig. 2b). The complete description of the identified allergenic proteins is compiled in Supplementary Table S4. Examples of the most relevant allergenic protein families identified are defensin-like proteins, lipid transfer proteins, pectate lyase, profilins, and polcalcins. Using sera of weed pollen allergic patients, IgE reactive spots confirmed the presence of the allergenic proteins identified by mass spectrometry (Supplementary Fig. S3). In addition to the information on the presence of members of known allergenic protein families, results on the entire proteome of feverfew pollen were obtained. After data refinement, where unknown products and redundant proteins were excluded, a total of 258 unique proteins were identified and annotated by Blast2GO software to gene ontology (Fig. 2a). Detailed information of gene ontology annotation regarding biological processes, cellular components and molecular functions are shown in Supplementary Fig. S2.

**Table 1:** Allergenic protein families identified in feverfew pollen by proteomics.

Identified allergenic protein families by (LC-MS/MS and 2-DE-LC-MS/MS)			
Allergenic protein family	LC-MS/MS	2D-LC-MS/MS	Spot #
60S acidic ribosomal protein	gi|729348440	gi|802562610	27
Aldehyde dehydrogenase	gi|8163730	gi|238846406	38
Aspartic peptidase (Peptidase A1 family)	gi|976909721	gi|976909721	10
Bet v 1 family	gi|976909362	gi|167472849	3
Beta-1.3-glucanase (Glycosyl hydrolase 17 family)	gi|976496731	gi|976928968	30
Calreticulin	gi|976913141	gi|976913141	9
Cupin family (Germin-like protein family)	gi|976899679	gi|976927409	18
Cu-Zn superoxide dismutase	gi|976902802	gi|3914999	48
Cyclophilin	gi|488726160	gi|332806715	2
Cysteine protease	gi|1706276	gi|28804503	11
Cytochrome c family	gi|118012	gi|976899814	17
Defensin-like protein	gi|817033923	gi|817033923	1
Enolase	gi|3023685	gi|976924044	30
Glyceraldehyde-3-phosphate dehydrogenase	gi|752855788	gi|902550458	33
Heat shock protein 70	gi|698946330	gi|976917633	9
LDH/MDH superfamily	gi|976916739	gi|976905497	32
NAC-alpha family	gi|729420144	gi|976923178	6
Pathogenesis-related protein PR-1	gi|743838314	gi|976917157	51
Pectate lyase	gi|113476	gi|302127818	39
Thaumatin-like protein	gi|20385169	gi|20385169	1
Thioredoxin	gi|720041636	gi|976899454	36
Triosephosphate-isomerase	gi|976918965	gi|976923382	1
Tubulin family	gi|803378277	gi|902550499	49
Chitinase	gi|425886502	n.i.	—
Chlorophyll a-b binding protein	gi|146403796	n.i.	—
Cobalamin-independent methionine synthase	gi|976902600	n.i.	—
Flavodoxin	gi|720077294	n.i.	—
Glutathione S-transferase	gi|698502214	n.i.	—
GMC oxidoreductase	gi|659069842	n.i.	—
Heat shock protein 90	gi|702435110	n.i.	—
Iron/manganese superoxide dismutase	gi|15551753	n.i.	—
Isoflavone reductase	gi|923534270	n.i.	—
L3 Ribosomal protein	gi|901818290	n.i.	—
Lipid transfer protein	gi|118490068	n.i.	—
Pectin methylesterase	gi|976571059	n.i.	—
Peroxiredoxin	gi|823226051	n.i.	—
Polcalcin	gi|976896497	n.i.	—
Polygalacturonase (Glycosyl hydrolase 28 family)	gi|976926788	n.i.	—
Profilin	gi|62249502	n.i.	—
Protein kinase	gi|976914952	n.i.	—
Pyrophosphatase	gi|901817400	n.i.	—
Serine protease (Peptidase S8 family)	gi|976923421	n.i.	—
Alcohol dehydrogenase	n.i.	gi|970021885	39
Beta-xylosidase	n.i.	gi|85813770	30
Endochitinase (Hevein like-domain)	n.i.	gi|848864792	9
Serine carboxypeptidase (Peptidase S10 family)	n.i.	gi|976807531	11
Xyloglucan endotransglucosylase (Glycosyl hydrolases family 16)	n.i.	gi|82394883	9

n.i., not identified.

### IgE reactivity to feverfew pollen extract and purified Par h 1

**Figure 3:**
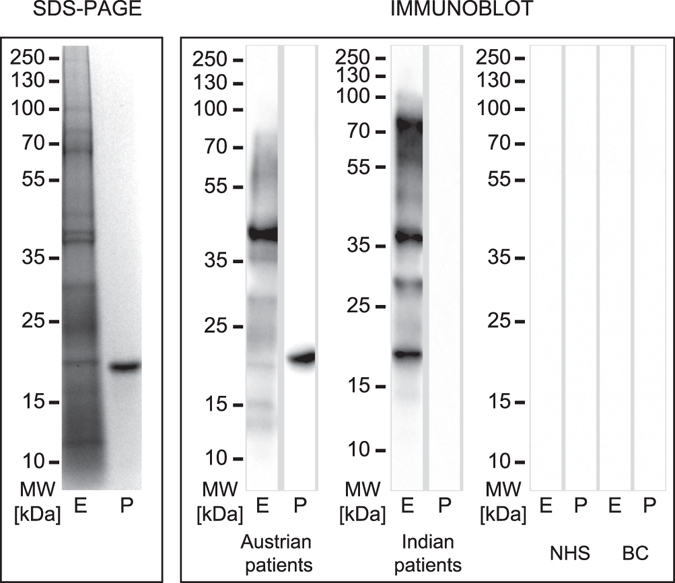
Immunoblot analysis of feverfew pollen extract (E) and purified recombinant Par h 1 (P). Extract and Par h 1 were loaded on reducing SDS-PAGE and stained with Coomassie. Immunoblot analysis was performed with a serum pool of Austrian weed pollen allergic patients (n = 15) and Indian feverfew pollen sensitized patients (n = 25). The unprocessed gels and immunoblots are shown in the Related Manuscript File. NHS, non-atopic human sera (n = 2) and BC, buffer control.

The allergenic protein profile of feverfew pollen extract and purified recombinant Par h 1 was analyzed by immunoblot using pooled sera from Austria and India (Fig. 3). IgE reactive proteins ranging from 15–100 kDa were detected using sera from both cohorts. Both groups of patients demonstrated solid IgE reactivity with a protein migrating around 40 kDa. Indian patients showed additional reactivity to a 20 kDa protein as well as high molecular weight proteins around 70–100 kDa. Natural glycosylated Par h 1 could be detected around 25 kDa in the extract, while the recombinant non-glycosylated molecule migrated at 20 kDa. Although IgE reactivity to the natural molecule was shown in both cohorts, the recombinant molecule was solely detectable with Austrian patients’ sera (Fig. 3).

**Figure 4:**
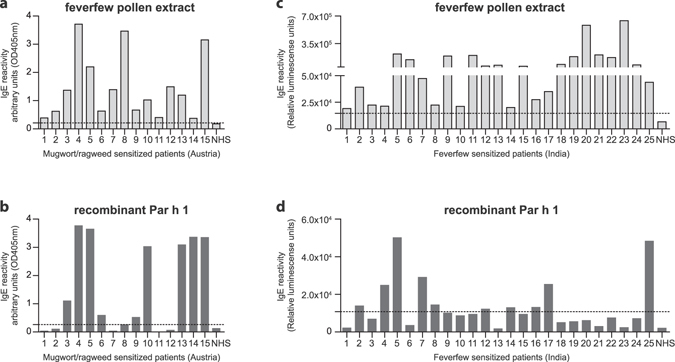
Feverfew extract and recombinant Par h 1 are recognized by patients’ sera in ELISA. IgE reactivity to immobilized feverfew pollen extract and purified recombinant Par h 1 was assessed with individual patients’ sera from Austria (**a**,**b**) and India (**c**,**d**). NHS, non-atopic human sera (n = 3). The dotted line indicates the response threshold calculated as 5*SD of the buffer control signal.

In addition, the IgE reactivity to feverfew pollen extract and Par h 1 was verified in ELISA using individual patients’ sera from fifteen Austrian weed pollen allergic patients and twenty-five Indian patients sensitized to feverfew pollen (Fig. 4). In the Austrian cohort, nine patients showed medium/high and six demonstrated lower IgE reactivity with feverfew extract. The recombinant molecule was recognized by six patients with medium/high and three with low IgE reactivity translating into a sensitization frequency of 60% in the tested group (Fig. 4a,b). Among Indian patients’ sensitized to feverfew pollen (n = 25), fourteen demonstrated high and eleven medium/low IgE reactivity towards the extract. Recombinant Par h 1 was recognized with good intensity by five patients’ sera and with lower signals by five other sera (Fig. 4c,d). The sensitization prevalence to Par h 1 was 40% among Indian feverfew positive patients.

### Feverfew pollen proteins inhibit IgE reactivity to allergens from mugwort and ragweed

**Figure 5:**
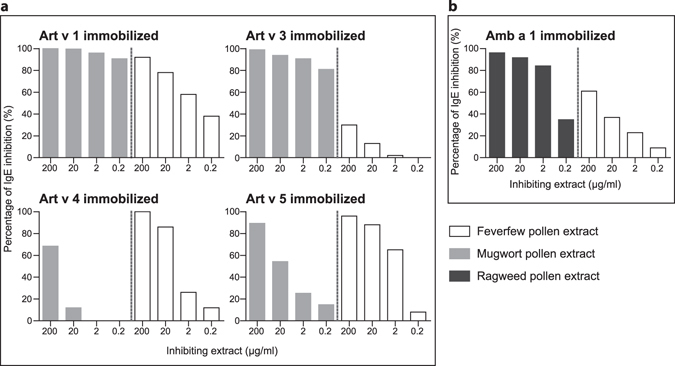
Feverfew pollen inhibits IgE reactivity to allergens from mugwort and ragweed. Purified weed pollen allergens were immobilized on ELISA plates. A pool of patients’ sera (n = 6) was pre-incubated with different concentrations of (**a**), mugwort (self-inhibition control) and feverfew pollen extract or (**b**), ragweed (self-inhibition control) and feverfew pollen extract. The sensitization profile to the single allergens of the patients’ pool is shown in the Supplementary Table S2.

In order to verify data obtained by mass spectrometry, the presence of potential allergens as well as IgE cross-reactivity was tested in an ELISA inhibition assay. Therefore, IgE binding against relevant allergens from mugwort and ragweed pollen was inhibited with different concentrations of feverfew pollen extract which indicates presence and cross-reactivity of such allergens in feverfew pollen (Fig. 5). For this experiment, a pool of patients’ sera (n = 6) with established IgE reactivity to tested mugwort and ragweed allergens was used. Feverfew pollen extract inhibited the IgE binding of Art v 1 (defensin-like protein), Art v 3 (LTP), Art v 4 (profilin) and Art v 5 (polcalcin) from mugwort in a dose-dependent manner, although at varying levels (Fig. 5a). Similarly, the IgE binding of the pectate lyase Amb a 1 from ragweed was inhibited (Fig. 5b). The specificity of inhibition was verified using unrelated inhibitors (BSA and FCS) and non-specific inhibition was ≤10%.

## Discussion

Allergic rhinitis to feverfew pollen has been documented for more than 25 years. Nevertheless, epidemiologic data as well as identification of allergenic proteins in this source have remained elusive. The only allergen described from this source and suggested to be a major allergen is a hydroxyproline-rich protein, from which only a partial amino acid sequence was retrieved^12^. In this work, we identified the full-length cDNA sequence of the corresponding protein using degenerated primer followed by 5′ RLM-RACE. The full-length sequence was subsequently officially acknowledged as Par h 1.0101 by the IUIS allergen nomenclature sub-committee. The cDNA sequence translates to a mature protein consisting of an N-terminal defensin-like domain and C-terminal proline-rich region. The N-terminal domain of the mature protein is in full concordance with the previously reported residues of the natural allergen^12^. The primary structure of the Par h 1 defensin-like domain is highly homologous to other plant defensin-proline fusion proteins all belonging to the Asteraceae family, e.g. Art v 1 from mugwort, Amb a 4 from ragweed and SF18 from sunflower pollen. In addition, the defensin-like domain of Par h 1 is similar to defensin proteins from other botanical families which do not contain a proline-rich region, e. g. Ah-AMP from horse chestnut (58%) and Rs-AFP 1 from radish (42%)^20^. The defensin-like domain of Par h 1 shows key features common to the defensin-like protein family like the cysteine pattern and several other conserved amino acid positions. The C-terminal domain presents a proline-rich region that differs in length and amino acid composition from homologs. For example, the C-terminal region of Par h 1 is 22 amino acids longer, and has a higher content of glycine, aspartic acid and glutamic acid residues than that of Art v 1 while it seems to be in general more similar to Amb a 4 and SF18 from sunflower. In the natural molecule, the C-terminal region of Par h 1 was reported to be partially hydroxylated^12, 19^. Exact localization and specific glycan determination needs however further investigation.

The mature protein sequence of Par h 1 was cloned and a recombinant molecule expressed as non-fusion protein in *E. coli*. The identity of the purified protein was unambiguously confirmed by intact mass analysis. A mass difference of 8.06 between reduced and unreduced Par h 1 indicated that all eight cysteine residues are involved in disulfide bridge formation. In SDS-PAGE analysis, the recombinant protein migrated at around 20 kDa, which is higher than the determined mass. This migration behavior is common for defensin-proline fusion proteins, has been also observed for Art v 1 and Amb a 4 and is likely due to the unordered poly-proline tail^20, 21^. When Par h 1 was subjected to CD measurements, a typical spectrum of polyproline-rich proteins was obtained, which was also very similar to purified natural and recombinant Art v 1^20, 22^. Additionally, only minor changes were observed upon thermal denaturation, i. e. a change in the spectrum from 220–230 nm and a small shift of 2 nm in the minimum. After the renaturation at 20 °C, the changes were partially restored and the spectrum at 220–230 nm regained the same characteristics as the native protein’s spectrum, while a different shape was obtained around the minimum position (198–205 nm). These results suggests that recombinant Par h 1 is able to partially recover the folding after thermal denaturation followed by renaturation, and are in agreement with results previously observed for Art v 1^22^.

Additional IgE reactive proteins from feverfew pollen were described 20 years ago by immunoblotting^11^. Hitherto, no further information on allergenic components of feverfew pollen is available, thus we decided to characterize the source in depth by proteomic analyses. We employed two complementary approaches to study the pollen proteome, LC-MS/MS and 2-DE-LC-MS/MS. For the proteomic study we decided on the combination of two strategies due to the advantages specific to each approach^23, 24^. Using the direct digestion of the pollen extract followed by LC-MS/MS, we were able to obtain higher numbers of proteins compared to the 2-DE-LC-MS/MS. However, with the preceding protein separation by 2-DE, we gained additional information on identified proteins such as molecular weight and isoelectric point. Additionally, sequencing of single protein spots increased the dynamic range when analyzing protein mixtures (ratio of lowest to highest abundance protein detectable), as peptides produced by in-gel tryptic digestion of each spot are sequenced in separate experiments^23^. Taking into account the before mentioned advantage for 2-DE-LC-MS/MS, we decided to use a higher cutoff (≥3 unique peptides) for this approach compared to LC-MS/MS (≥1 unique peptide) for a valid identification hit. A grand total of 258 protein species were identified in the pollen preparation by these two strategies. From these proteins, 47 hits were previously identified to represent allergenic protein families according to IUIS allergen nomenclature sub-committee database entries. The presence of Par h 1 was confirmed by both proteomics strategies. In 2-DE-LC-MS/MS, Par h 1 was identified in seven spots (Fig. 2b, spots 1–7). This finding suggests the presence of other isoforms not yet identified as well as different post-translational modification. The result is in line with the initial study identifying three variants of Par h 1 with different isoelectric points^12^. Additionally, we observed the presence of several allergenic protein families commonly found in pollen of weeds, grasses and trees^25^. This included e. g. pectate lyases lipid-transfer proteins, profilins, polcalcins, cysteine proteases, polygalacturonases, and enolases. Our results are in accordance with other studies where some of the before mentioned protein families have been identified using proteomics approaches in plants botanically related to feverfew. A recent work identified the presence of IgE reactive pectate lyase and cysteine protease in sunflower using 2-DE electrophoresis analysis^26^. In ragweed, transcriptome and immunoproteome studies revealed the presence of a cysteine protease (Amb a 11), polygalacturonase and enolase^27, 28^. We also identified a protein belonging to the PR-10 protein family which might most likely be explained by minute contaminations with pollen grains from oak and birch.

In this study we also provided for the first time a proteomic profile of feverfew pollen. We identified a group of pollen-specific protein families, e. g. germin-like protein family, glycosyl hydrolase family and actin, that have been also identified in the pollen of *Arabidopsis thaliana* by proteomics^29^. Our study is in concordance with other pollen proteomics and transcriptomics study where most of the proteins were annotated to metabolic processes, cellular processes, single organism processes and response to stimuli^30, 31^. Thus, we elucidated distribution of proteins involved in biological process, cellular component and molecular function that can be helpful for further studies with this plant (Supplementary Fig. S2).

To evaluate the presence of IgE reactive molecules in the extract we used sera from Austrian weed pollen and Indian feverfew pollen sensitized patients. Using serum pools, our immunoblot showed a broad range of reactivity which was however covering a larger molecular weight range compared to previous studies^11, 32^. Both, Austrian and Indian patients reacted strongly to a 40 kDa protein corresponding to a pectate lyase as shown by mass spectrometry and IgG reactivity with an Amb a 1-specific antibody (Supplementary Fig. S4a). Interestingly, Indian sera reacted stronger to a band migrating slightly below the one reactive with the Austrian sera which might relate to different isoform recognition as previously revealed for Amb a 1 isoforms^28^. Natural Par h 1 was detected by the Austrian and Indian serum pool, even though reactivity was diffuse due to heterogeneity of the glycosylated molecule. The recombinant molecule was only recognized by the Austrian sera, which might be due to excessive dilution of Par h 1-specific antibodies in the Indian serum pool and/or recognition of discontinuous IgE epitopes affected by reducing conditions in gel electrophoresis. Notably, Indian patients showed strong reactivity with a 20 kDa and 70–80 kDa protein which could - according to our mass spectrometry data - correspond to a PR-1 protein and thioredoxin, respectively. Indeed, the PR-1 protein Art v 2 was previously described as mugwort allergen with a sensitization prevalence of 58%^33^. Further studies are needed to clarify the relevance of these IgE binding proteins in a molecule-based approach.

IgE reactivity to feverfew extract and purified recombinant Par h 1 was tested in ELISA using individual sera from Austrian and Indian patients. As mentioned above, feverfew pollen is neither standardized nor included in routine allergy diagnosis even though it represents a major health issue in some regions. Therefore, our inclusion criteria for Indian patients were broader taking into account allergic patients who were screened for sensitization to feverfew extract. This circumstance might also explain why IgE levels were generally lower in this cohort. Recombinant Par h 1 was recognized by 60 and 40% of Austrian and Indian sera, respectively. Our data revealed that IgE epitopes are present in the polypeptide backbone, since recombinant Par h 1 is non-glycosylated due to production in *E. coli*. This result brings an additional understanding on the allergenicity of this protein, since previous reports suggested that IgE binding was depending on the carbohydrates moiety^12^. The IgE binding observed for Par h 1 using sera from mugwort and ragweed pollen sensitized patients from Austria supports previous studies observing cross-reactive symptoms between ragweed and feverfew pollen allergic patients^2^. The fact that IgE binding to Par h 1 is observed in Austrian patients who are typically not exposed to feverfew suggests the presence of cross-reactive IgE induced by primary mugwort and/or ragweed sensitization. In addition, the molecule was also recognized by Indian sera reactive to feverfew extract. Based on the fact that IgE cross-reactivity of extract and purified protein was not entirely overlapping and sensitization prevalence was between 40–60%, Par h 1 might not represent the major allergen of the source as previously suggested by Gupta *et al*.^12^. Nevertheless, the identification of the complete sequence of Par h 1 and its recombinant production have an important value for molecule-based cross-reactivity studies, since the presence of allergenic defensin-proline fusion proteins within the Asteraceae family is well documented^25^.

In ELISA inhibition assays, feverfew pollen extract inhibited IgE-binding to relevant pollen allergens of mugwort and ragweed. The presence of structurally-related and IgE cross-reactive allergens belonging to the defensin-like family, LTP, profilin, polcalcin and pectate lyase was confirmed. These results are in line with the IgE binding profile determined by immunoblotting as well as with mass spectrometry data. As an example, the presence of a pectate lyase matched with the finding of this family in the proteomic study and was further corroborated by the presence of a strongly IgE reactive band at 40 kDa in immunoblot. IgE cross-reactivity within the Asteraceae family has been documented before, e.g. the defensin-proline fusion proteins Art v 1 (mugwort) and Amb a 4 (ragweed)^21^ and the pectate lyase Art v 6 (mugwort) and Amb a 1 (ragweed)^17, 34^. Lower levels of IgE cross-reactivity were found with the LTP Art v 3, which could be due to either lower abundance or dissimilar primary sequence as also observed with Amb a 6 from ragweed pollen^35, 36^. LTPs present a wide range of sequence similarity and IgE cross-reactivity which are additionally highly patient specific^37–39^. However, the presence of an Art v 3 cross-reactive LTP could be unambiguously verified by immunoblot analysis (Supplementary Fig. S4b).

In summary, a comprehensive study of the protein profile of feverfew pollen was performed and a panel of molecules belonging to different allergenic protein families was identified. The proteomic results were further validated by immunoblot and inhibition ELISA, where IgE cross-reactivity to clinically relevant allergens was confirmed. We cloned and produced recombinant Par h 1, an allergenic defensin-proline fusion protein and our molecule-based approach should help to further investigate the cross-reactivity of defensin-like proteins within allergenic Asteraceae pollen. Feverfew is now showing a tremendous geographic distribution, while based on predicted climatic changes this weed might become a strong global player in allergy in the future. Based on our data we suggest the inclusion of standardized feverfew (*Parthenium hysterophorus)* extract in routine allergy diagnosis for countries where the weed is endemic.

## Electronic supplementary material

Supplementary Information

Supplementary Table S3
