# Supplementary material for: Proteomic profiling of the weed feverfew, a neglected pollen allergen source
Source: Sci Rep. 2017 Jul 20;7:6049. doi: 10.1038/s41598-017-06213-z (PMC5519751; doi:10.1038/s41598-017-06213-z)
Supplement: Supplementary file 1 — Supplementary Information [file 41598_2017_6213_MOESM1_ESM.pdf]

Proteomic profiling of the weed feverfew, a neglected pollen allergen source

Isabel Pablos[1], Stephanie Eichhorn[1], Peter Briza[1], Claudia Asam[1], Ulrike Gartner[2], Martin Wolf[1], Christof Ebner[3], Barbara Bohle[4], Naveen Arora[5], Stefan Vieths[6], Fatima Ferreira[1], Gabriele Gadermaier[1]

[1]University of Salzburg, Department of Molecular Biology, Division of Allergy and Immunology, Salzburg, Austria

[2]University of Salzburg, Department of Ecology and Evolution, Salzburg, Austria

[3]Allergy Clinic Reumannplatz, Vienna, Austria

[4]Department of Pathophysiology and Allergy Research, Medical University of Vienna, Vienna, Austria

[5]CSIR-Institute of Genomic and Integrative Biology, Allergy and Immunology Section, Delhi, India

[6]Paul-Ehrlich-Institut, Federal Institute for Vaccines and Biomedicines, Langen, Germany

Corresponding author

Dr. Gabriele Gadermaier

Department of Molecular Biology, University of Salzburg

Hellbrunnerstraße 34, A-5020 Salzburg, Austria

Telephone: 0043-662-8044-5974, Fax: 0043-662-8044-183

Gabriele.Gadermaier@sbg.ac.at

ONLINE MATERIAL AND METHODS

Patients' sera

Weed pollen-allergic individuals (n=15) were selected on the basis of typical case history, i.e. recurrent rhinitis/conjunctivitis during late summer, and allergen-specific IgE determined by ImmunoCAP (Thermo Fisher Scientific, Uppsala, Sweden) to mugwort (w6) and ragweed (w1) pollen (Table S1). For the inhibition experiment, a pool of six patients was used covering sensitization profiles to Art v 1, Art v 3, Art v 4, Art v 5 and Amb a 1 (Table S2). In addition, we screened forty-four Indian sera from allergic patients for IgE reactivity to feverfew pollen. Information on patients with positive reactivity (n=25) is shown in the Table S1. Experiments using anonymized serum samples from allergic patients were approved by the ethics committee of the Medical University of Vienna (712/2010) and the CSIR-Institute of Genomics and Integrative Biology, New Delhi, India (CLP 0019). Informed written consents were obtained from all subjects.

Pollen extracts

The inflorescence of *Parthenium hysterophorus* was collected in the month of April from Delhi. It was freeze-dried until completely dry. This material was sieved through 100, 200 and 300 micron nylon mesh to remove unwanted plant parts and debris. The final purity of sieved pollen grains were determined by staining with safranin and observed under the microscope (40X). About 1000 pollen in different microscopic fields were counted to determine the purity. The impurity contained plant/floral parts, dust particles, other pollen and fungal spores. The pollen grains used for the present study had >97% purity. In Austria, feverfew pollen grains were transferred on a microscope slide and stained with safranin to be able to distinct pollen from other plant material. The preparations were analyzed and counted using a Reichert Diavar microscope. For the preparation of protein extracts, 1 g of feverfew pollen was dissolved in 10 mL of 10 mM dipotassium phosphate pH 7.0, 2 mM ethylenediaminetetraacetic acid and 2% (w/v) polyvinylpolypyrrolidone and shaken overnight at 4°C. Afterwards the solution was centrifuged at 40,000 g for 60 minutes and the supernatant was dialyzed to 5 mM of sodium phosphate pH 7.0 overnight at 4 °C. The protein extract was stored at -20 °C until

further use. Pollen grains from, mugwort *(Artemisia vulgaris)* and ragweed *(Ambrosia artemisiifolia)* were purchased from Allergon AB (Ängelholm, Sweden) and the pollen extracts were prepared following Gadermaier *et al.* [1].

cDNA cloning of Par h 1

Pollen grains from feverfew were ground and total RNA purification was performed using TRIzol (Ambion/RNA, Invitogen, Carlsbad, CA, USA). The reverse transcription was achieved using oligo dT primer and the Super-Script III First-strand Synthesis System (Invitrogen). Amplification of the cDNA was obtained after nested PCR with forward degenerated primers (5'-TGGTTYGGNAAYTGYAARGA-3' and 5'-TGYAARGAYACNGARAARTGYGA-3') based on the partial Par h 1 amino acid sequence previously described [2]. The amplified products were cloned into the pGEM-T Easy Vector (Promega, Madison, WI, USA). The 5'-UTR and the signal peptide sequence were identified using the 5' RLM-RACE protocol and the gene specific primers 5'-GGACCTGGATTCTTCTTAGGATCA-3' and 5'-CTTAGGATCACAATCAAAGTAGC-3'. The C-terminal part of the sequence was obtained using a forward gene specific primer localized in the signal peptide 5'-ATGGCGAAGAGTTCAACTTCTTACTTAGT-3' and oligo dT.

Production of recombinant allergens

Par h 1 mature protein sequence was cloned into pHisParallel2 vector using 5'-GAGACATATGGGTAAAGTATGTGA-3' as forward primer, and 5'-TCTCCTCGAGCTAAGCAGCTGGTG-3' as reverse primer with *Nde*I and *Xho*I restriction sites respectively. In order to increase the stability during expression, a glycine residue was introduced at the N-terminus. Recombinant Par h 1 was expressed as non-fusion protein in *E. coli* Rosetta-gami B (DE3) pLysS (Novagen, Gibbstown, NJ, USA). The bacterial culture was induced with 0.4 mM isopropyl-b-D-thiogalactopyranoside and grown at 20 °C for 16 hours. Bacterial cells were harvested by centrifugation at 4000g for 20 minutes. Cells were lysed by cycles of freezing and thawing followed by sonication (2 min, pulsing 3x at 50%) in lysis buffer; 10 mM sodium phosphate pH 7.0, 300 mM sodium chloride and 2.5 µg/mL DNAse final concentrations. Lysate supernatants were

precipitated stepwise with ammonium sulfate at 40% of saturation (1.6 M) at 4 °C. Par h 1 enriched supernatant obtained after the protein precipitation, was subjected to two steps of hydrophobic interaction chromatography using 10 mM sodium phosphate pH 7.0, 300 mM sodium chloride and ammonium sulfate 1.6 M as binding buffer and 10 mM sodium phosphate pH 7.0, 10 mM sodium chloride and 8% isopropanol as elution buffer. First, the protein solution was passed through 1 mL low substitution phenyl column using 20% of the elution buffer and unbound proteins were subsequently loaded onto 5 mL high substitution phenyl column. Proteins were then eluted using an increasing gradient of elution buffer, 20-100% in 60 min at a flow rate of 1 mL/min. Fractions containing Par h 1 were pooled and the final purification was performed by size exclusion using a Superdex 75 10/300 GL column (GE Healthcare, Little Chalfont, UK) in 5 mM of ammonium carbonate pH 7.8 at a flow rate of 0.3 mL/min. The purified protein was stored at -20 °C until further use.

Art v 1 from mugwort was expressed as described above for Par h 1 and bacterial lysate were obtained in the same way. The lysis buffer for Art v 1 was 10 mM ammonium hydrogen carbonate pH 7.8, 10 mM sodium chloride and 2.5µg/mL DNAse final concentration. Soluble fraction containing Art v 1 was ultra-filtrated through a membrane with 50 kDa cut-off (Vivacell 250 insert, Sartorius, Germany). The ultrafiltration process was followed by one step of cation exchange chromatography (SP Sepharose Fast Flow, (GE Healthcare) where 50 mM sodium acetate pH 5.35 was the binding condition and for elution sodium chloride concentration was gradually increased to 1M. Fractions containing Art v 1 were pooled and the final purification was performed by size exclusion using a Superdex 75 10/300 GL column (GE Healthcare) in 5 mM of ammonium carbonate pH 7.8 at a flow rate of 0.3 mL/min. The purified protein was stored at -20 °C until further use. Recombinant purified allergens from mugwort Art v 3, Art v 4 and Art v 5 were obtained as described [3-5]. Natural Amb a 1 from ragweed was obtained as previously described [6].

Physicochemical characterization of purified Par h 1

Amino acid analysis was performed with the protein in duplicates following the Pico-Tag™ method (Waters, Milford, MA, USA). Phenylthiocarbamyl amino acid derivatives were analyzed by reversed

phase high-performance liquid chromatography (UltiMate 3000, Thermo Fischer, Waltham, MA, USA), using a 3.0x150 mm XSELECT™ HSS T3 3.5 µm column (Waters). Hydrolyzed amino acid peaks were quantified at 254 nm by peak area comparison to amino acid standard H (Pierce, Rockford, IL, USA).

For intact mass measurements of purified Par h 1, the sample was desalted with C_{18} ZipTips (Merck, Millipore, Billerica, MA, USA) and directly infused into the Q-Exactive mass spectrometer (Thermo Fisher Scientific) at a flow rate of 1 µl/min, using the nano electrospray head. Raw data obtained from intact proteins were processed with Protein Deconvolution 2.0 (Thermo Fisher Scientific).

Circular dichroism spectra to study the secondary structure were recorded in 10 mM of potassium phosphate pH 7.0 with a JASCO J-815 spectropolarimeter (Jasco, Tokyo, Japan). Spectra were also recorded when the protein was heated up to 95 °C and cooled down to 20 °C. Far UV spectra (190–260 nm) were baseline subtracted and results are presented as the mean residue molar ellipticity.

1D and 2D-gel electrophoresis

Feverfew pollen extract, bacterial lysates and purified Par h 1 were analyzed by reducing polyacrylamide gel electrophoresis using 15% gels. Proteins were visualized with Coomassie Brilliant Blue R-250 staining (Bio-Rad, Hercules, CA, USA). For 2D gel electrophoresis, 30mg of feverfew pollen grains were ground to fine powder in liquid nitrogen and the pollen extract was obtained as described [7]. The vacuum dried pellet was re-suspended in 300 µL of isoelectric focusing buffer and proteins were loaded into ReadyStrip IPG strip pH 3-10 (Bio-Rad Laboratories) by rehydration at 50 mA for 16 hours at room temperature. The buffer compositions and the isoelectric focusing conditions are the same as described by [1]. Afterwards, the strip was loaded onto 15% acrylamide gels, and proteins were separated by reducing SDS-PAGE. Proteins were first visualized with Coomassie Brilliant Blue R-250 (Bio-Rad Laboratories) and the obtained spots were excised from gels for further use.

Mass spectrometry of feverfew proteins

For proteomic analyses of feverfew pollen, two complementary methods were used. The first approach relied on generating a tryptic digest of the pollen extract which was directly analyzed by reverse-phase liquid chromatography mass spectrometry (LC-MS/MS). In this case the protein extract from 30 mg of pollen was prepared using the same extraction method described by [7]. The other method consisted in separation of the pollen proteins by two-dimensional electrophoresis (2-DE), as described above, followed by in-gel digestion of the protein spots and mass analysis (2-DE-LC-MS/MS). Both preparation, coming from the gel spots or directly from the extract, were reduced, alkylated and digested with ProteoExtract All-in-One Trypsin Digestion Kit (Merck Millipore). Resulting peptides were separated by reverse-phase nano-HPLC (Dionex Ultimate 3000, Thermo Fisher Scientific, Bremen, Germany). Peptides were loaded onto the trap column (PepSwift Monolithic Trap Column, Dionex) and desalted with 0.1% (v/v) heptafluorobutyric acid at a flow rate of 10 µl/min. After 5 minutes, trap and separation column (PepSwift Monolithic Nano Column, 100 µm x 25 cm, Dionex) were coupled with a switching valve and the peptides were eluted with an acetonitrile gradient (Solvent A: 0.1% (v/v) formic acid, 0.01% (v/v) trifluoroacetic acid, 5% (v/v) acetonitrile; solvent B: 0.1% (v/v) formic acid, 0.01% (v/v) trifluoroacetic acid, 90% (v/v) acetonitrile; 5–45% B in 60 min) at flow rate of 1 µl/min at 55 °C. The HPLC was directly coupled via nano electrospray to a Q-Exactive mass spectrometer (Thermo Fisher Scientific). Capillary voltage was 2 kV. For peptide identification, a top 12 method was used; instrument settings were based on the 'sensitive' method described by [8], with only minor modifications. The normalized fragmentation energy was 28%. Survey and fragment spectra were analyzed with Proteome Discoverer version 1.4 (Thermo Fisher Scientific) or Peaks Studio 7.5 (Bioinformatics Solutions, Waterloo, Canada), respectively.

Database search and Gene Ontology annotations

Fragment spectra obtained from LC-MS/MS experiments were searched against the NCBInr database with PEAKS Studio 7.5. A target-decoy search strategy was employed and the false discovery rate (FDR) was calculated by the decoy-fusion method. Protein scores were set based on the desired FDR of 1%. Identifications were only considered when at least one unique peptide, for in-solution digested

samples or three for in-gel digested samples were present. Additionally, the data were manually inspected, proteins identified with the same group of peptides were merged with the one with the higher score, and the amino acids leucine and isoleucine were considered as equal. Afterwards, proteins with unknown description were also excluded from the analysis in both methods.

In order to eliminate double identifications arising from highly conserved proteins across plant species, a protein blast strategy was performed as follows. The list of all proteins identified either by LC-MS/MS or 2-DE-LC-MS/MS was compared to itself. Those proteins with an identity score higher than 100 were considered to be the same hit and therefore only the protein with the highest identification score in MS was kept in the list. Each of these lists identified by the two strategies, LC-MS/MS and 2-DE-LC-MS/MS, was further compared to the Allergome database (uniprot release 29.03.2016) and an identity score of ≥ 50% was set for allergenic proteins. Besides, the obtained allergenic families where further inspected according to the International Union of the Immunological Societies (IUIS) for allergen nomenclature sub-committee. In summary, an identified protein was considered an allergenic protein when the homology with a known allergen gave an identity score ≥ 50% (according to Allergome) and was officially recognized by the IUIS allergen nomenclature sub-committee.

In order to describe the entire proteome of feverfew pollen a final list of proteins was created by eliminating redundant proteins. The final list of proteins was annotated to Gene Ontology using the commercially available software Blast2GO. Pie chart graphs were created for biological process with level 2, cellular component with level 2 and molecular function with level 3.

Immunoblot, ELISA and cross-inhibition experiments

For immunoblots, feverfew pollen extract and purified Par h 1 were first separated by SDS-PAGE and then proteins were electroblotted onto nitrocellulose membrane (Amersham, GE Healthcare Life Science, Germany). Serum pools of weed pollen allergic patients from Austria (n=15) and feverfew sensitized patients from India (n=25) were diluted 1:10 in blocking buffer (25 mM Tris pH 7.5, 0.15 M

sodium chloride, 0.5% (v/v) Tween 20, 0.5% (w/v) bovine serum albumin, and 0.05% (w/v) sodium azide) and incubated overnight at 4 °C. Alternatively for identification of homologous allergens, non-specific lipid transfer protein and pectate lyase, monoclonal and polyclonal antibodies were used as primary antibodies. In specific, mouse monoclonal antibodies raised against recombinant Art v 3.0201 (unpublished data from our laboratory) and rabbit polyclonal antibodies raised against natural Amb a 1 (kindly provided by T.P. King) were used. For detection of IgE in immunoblot, a horseradish peroxidase-conjugated monoclonal anti-human IgE, diluted 1:100000, (Southern Biotech, Birmingham, AL, USA) was used as secondary antibody. The signal was detected using the chemiluminescence Amersham ECL-prime detection system (GE Healthcare Bio-Sciences AB Björkgatan, Sweden) according to manufacturer's protocol. For identification of homologous allergens, a rabbit anti-mouse IgG/IgM (Jackson ImmunoResearch) and a goat anti-rabbit IgG (Jackson ImmunoResearch, West Grove, PA, USA) alkaline phosphatase-conjugated were used as secondary antibodies, respectively. A colorimetric detection with nitro-blue tetrazolium and 5-bromo-4-chloro-3'-indolyphosphate (AppliChem, Darmstadt, Germany) was performed.

For ELISA experiments, 0.2 μg of feverfew pollen extract or purified Par h 1 were immobilized. Nunc-Immuno MaxiSorp high binding ELISA plates (Nunc, Roskilde, Denmark) were used for colorimetric and Lumitrac high binding ELISA plates (Greiner Bio-One, Germany) were used for chemiluminescence detection. ELISA plates were blocked with 137 mM sodium chloride, 10 mM Tris pH 7.5, 0.05% (v/v) Tween 20 (TBST) and 1% (w/v) BSA. Sera from 15 weed pollen allergic patients from Austria, 25 feverfew sensitized patients from India or healthy donors were diluted 1:5 in TBST and 0.5% (w/v) BSA and incubated on the plate overnight at 4 °C. For IgE detection, the alkaline phosphatase-conjugated anti-human IgE (BD Biosciences) for colorimetric system and the horseradish peroxidase-conjugated monoclonal anti-human IgE (Southern Biotech, Birmingham, AL, USA) for chemiluminescence system were used as secondary antibodies, respectively. According to the detection system, the signal was measured using the substrate 4-nitrophenyl phosphate (Sigma-Aldrich, St Louis, MO, USA) at OD of 405/492 nm or BM chemiluminescence ELISA substrate POD (Roche, Germany). The results were shown as arbitrary units with absorbance (OD 405nm) and the

relative chemiluminescent unit (RLU), respectively. The threshold response was calculated as 5*SD of the buffer control signal.

Additionally, direct ELISA experiments were performed for selection of sera reactive to mugwort and ragweed pollen allergens. IgE reactivity to purified recombinant Art v 1, Art v 3, Art v 4, Art v 5 and Amb a 1 was verified (Table S2). Purified Art v 1, Art v 3, Art v 4, Art v 5 and Amb a 1, were immobilized on Lumitrac high binding ELISA plates (Greiner Bio-One, Germany). For detection, the same protocol as described above was used.

For the IgE cross-inhibition experiments purified allergens, i.e. Art v 1, Art v 3, Art v 4, Art v 5 and Amb a 1 were immobilized on Lumitrac high binding ELISA plates (Greiner Bio-One). The serum pool described above and shown in Table S2 (n=6) was pre-incubated with increasing concentrations (0.2, 2, 20, 200 µg/mL) of pollen extracts from mugwort, ragweed, feverfew and buffer. The inhibition ELSA was validated using unrelated inhibitor proteins, i.e. BSA and fetal calf serum (FCS). IgE reactivity was visualized by chemiluminescence as described above and results are shown as percentage of inhibition.

REFERENCES

1 Gadermaier, G. et al. Plantago lanceolata: an important trigger of summer pollinosis with limited IgE cross-reactivity. The Journal of allergy and clinical immunology 134, 472-475, doi:10.1016/j.jaci.2014.02.016 (2014).

2 Gupta, N., Martin, B. M., Metcalfe, D. D. & Rao, P. V. Identification of a novel hydroxyproline-rich glycoprotein as the major allergen in Parthenium pollen. J Allergy Clin Immunol 98, 903-912 (1996).

3 Gadermaier, G. et al. Isoform identification and characterization of Art v 3, the lipid-transfer protein of mugwort pollen. Molecular immunology 46, 1919-1924, doi:10.1016/j.molimm.2009.03.021 (2009).

4 Wopfner, N. et al. Molecular and immunological characterization of profilin from mugwort pollen. Biological chemistry 383, 1779-1789, doi:10.1515/bc.2002.199 (2002).

5 Wopfner, N. et al. Molecular and immunological characterization of novel weed pollen pan-allergens. Allergy 63, 872-881, doi:10.1111/j.1398-9995.2008.01635.x (2008).

6 Pichler, U. et al. Pectate lyase pollen allergens: sensitization profiles and cross-reactivity pattern. PLoS One 10, e0120038, doi:10.1371/journal.pone.0120038 (2015).

7 Sheoran, I. S., Ross, A. R. S., Olson, D. J. H. & Sawhney, V. K. Compatibility of plant protein extraction methods with mass spectrometry for proteome analysis. Plant Science 176, 99-104, doi:http://dx.doi.org/10.1016/j.plantsci.2008.09.015 (2009).

8 Kelstrup, C. D., Young, C., Lavallee, R., Nielsen, M. L. & Olsen, J. V. Optimized fast and sensitive acquisition methods for shotgun proteomics on a quadrupole orbitrap mass spectrometer. Journal of proteome research 11, 3487-3497, doi:10.1021/pr3000249 (2012).

a

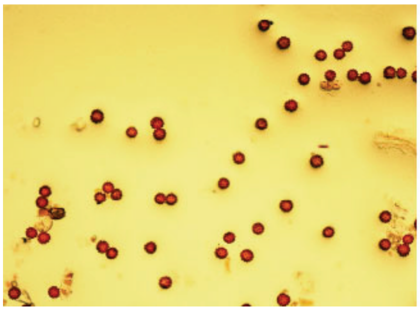

b

	Slide 1	Slide 2	Slide 3
Betula (birch)	1		1
Quercus (oak)	1	1	
Fraxinus (ash)		1	
Poaceae (grass)	1	1	3
Pteridophyta (fern)			1
Parthenium (feverfew)	997	997	995

Supplementary Fig S1: Quality assessment of feverfew pollen. **a**, Microscopy image of the pollen grains. **b**, Summary of three independent pollen counts using a Reichert Diavar microscope. Pollen grains were transferred on a microscope slide and stained with safranin.

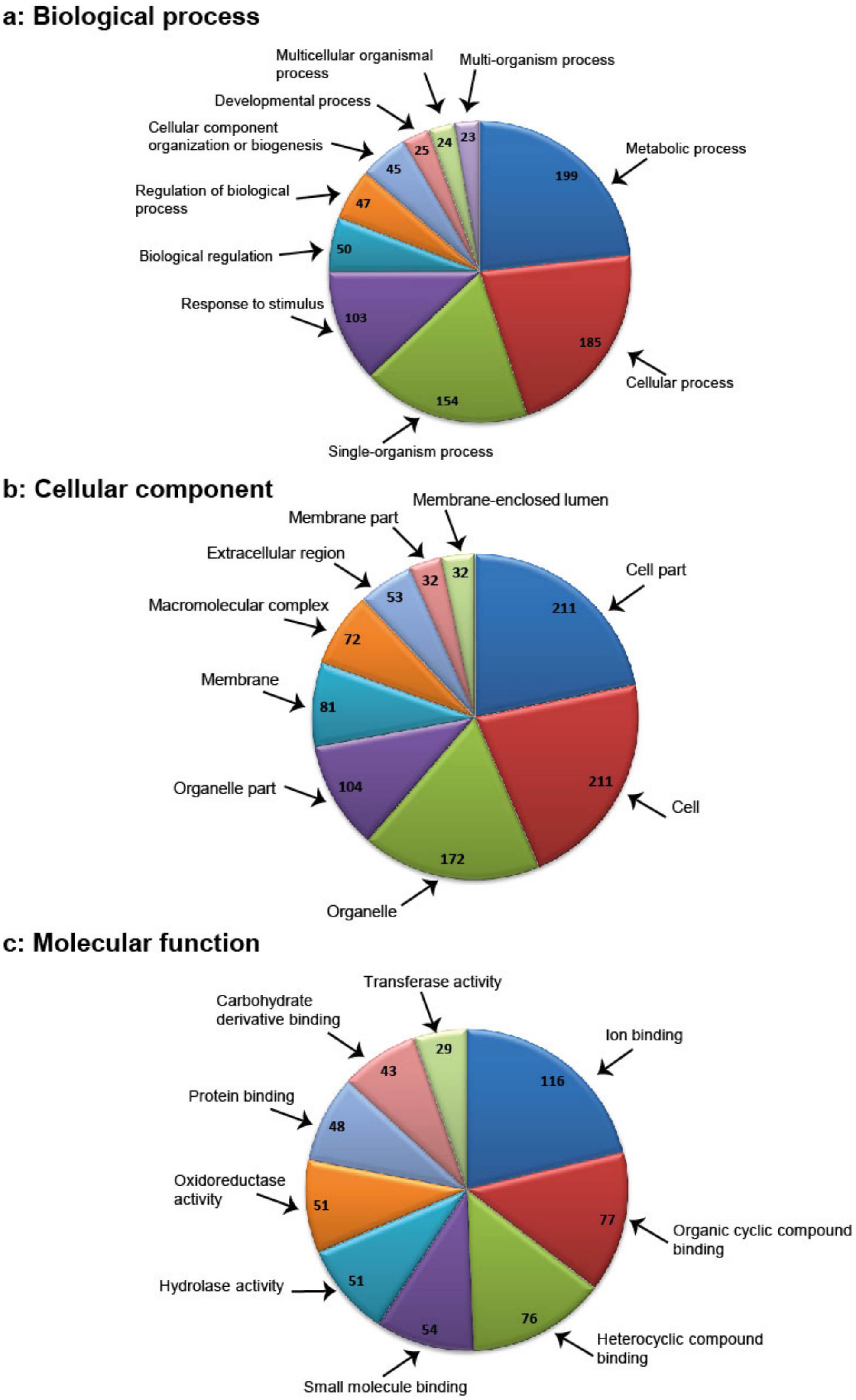

Supplementary Fig S2: Gene ontology annotation of identified proteins from feverfew pollen using Blast2GO software. A total of 258 proteins were annotated to **a,** biological process, **b,** cellular component, **c,** molecular function.

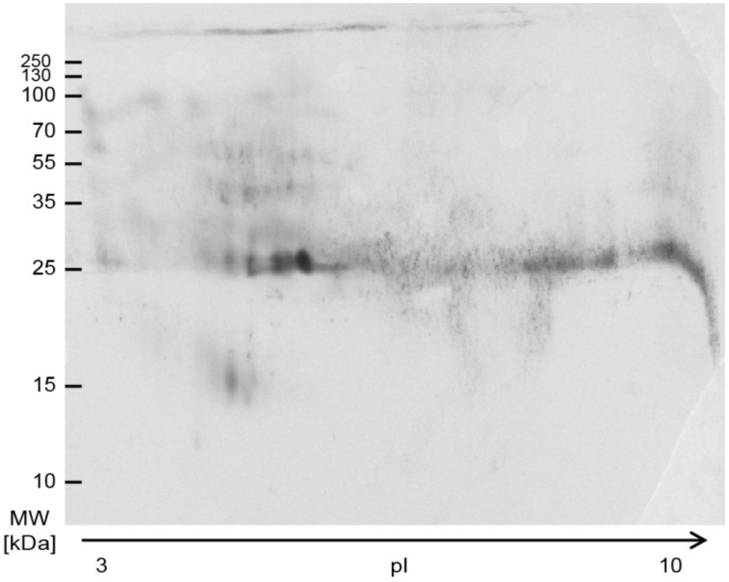

Supplementary Fig S3: Immunoblot analysis of feverfew extract separated by 2D gel electrophoresis. After 2D separation, IgE reactivity to feverfew pollen proteins was analyzed with a serum pool of Austrian patients.

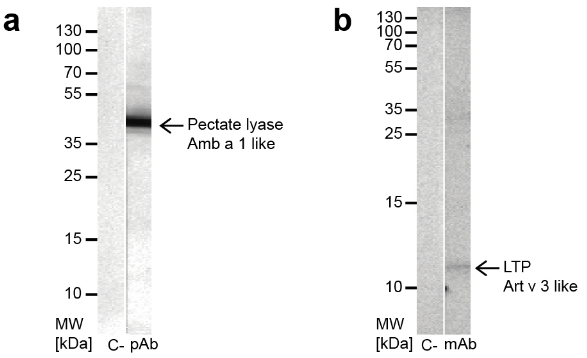

Supplementary Fig S4: Homologs of LTP and pectate lyase are present in feverfew pollen. Proteins from feverfew pollen extract were electroblotted onto nitrocellulose membrane and incubated with blocking buffer (negative control C-) or with, **a,** a murine monoclonal antibody raised against recombinant Art v 3.0201, the non-specific LTP from mugwort pollen and **b,** a rabbit polyclonal antibody raised against natural Amb a 1, the pectate lyase from ragweed pollen. Full-length blot is presented in Related Manuscript File 4 and 5.

Supplementary Table S1: Information on Austrian weed pollen allergic patients and Indian feverfew sensitized patients

Austrian patients	Gender	Age	Symptoms	RAST class Mugwort (w6)	RAST class Ragweed (w1)
A 1	M	18	Rhinitis/conjunctivitis	4	5
A 2	M	15	Rhinitis/conjunctivitis	5	6
A 3	M	22	Rhinitis/conjunctivitis	5	5
A 4	M	61	Rhinitis/conjunctivitis	5	5
A 5	F	39	Rhinitis/conjunctivitis	6	6
A 6	M	16	Rhinitis/conjunctivitis	4	5
A 7	M	49	Rhinitis/conjunctivitis	3	4
A 8	M	17	Rhinitis/conjunctivitis	4	5
A 9	F	44	Rhinitis/conjunctivitis	3	5
A 10	M	20	Rhinitis/conjunctivitis	5	6
A 11	M	57	Rhinitis/conjunctivitis	2	5
A 12	M	25	Rhinitis/conjunctivitis	3	5
A 13	F	69	Rhinitis/conjunctivitis	6	5
A 14	M	63	Rhinitis/conjunctivitis	3	5
A 15	M	16	Rhinitis/conjunctivitis	4	4

Indian patients	Gender	Age	Symptoms	Skin prick test
I 1	M	39	BA	Parthenium, Alternaria, Cynodon
I 2	M	43	AR	Parthenium, Cockroach, Prosopis
I 3	M	34	NBA	Cockroach, Parthenium
I 4	M	45	ND	ND
I 5	F	37	AR	Cockroach, Parthenium, Prosopis
I 6	M	48	AR	Prosopis, Eucalyptus, Gum Acacia
I 7	M	11	Vit D deficiency	Prosopis
I 8	M	52	NBA	Prosopis, Parthenium
I 9	M	26	Coeliac disorder, MODM	Prosopis, Wheat, Cockroach
I 10	F	35	AR	Cockroach, Prosopis
I 11	F	61	BA	Alternaria, Prosopis, Eucalyptus
I 12	M	26	ND	Chenopodium album, Grain dust, cockroach, Dust mite
I 13	M	26	AR	Prosopis
I 14	M	45	Angioedema, BA	Prosopis, Rhizopus, Cockroach
I 15	F	34	AR, U	Aspergillus fumigatus, Cockroach
I 16	M	45	AR	Cockroach, Pizzamix
I 17	M	30	ANB	Drumstick,Coconut fresh, Beanmix, Cockroach
I 18	M	18	AR	Cockroach, Prosopis, Imperata cylindr.
I 19	M	29	ND	Grain dust mix, Grain dust wheat, Agreatum conyzooides, Amaranthus, Holoptila integr., Cockroach
I 20	M	31	AR	Prosopis, Ricinus, Zea mays, Alternaria ten. Almond, Ground nut, Moth Dal, Patato, Cabbage, Cockroach
I 21	M	2	BA	Milk, Cockroach
I 22	M	29	ND	Moth, House fly, Epicoccum, Cockroach
I 23	M	28	ND	Moth, Mosquito, Cockroach
I 24	M	35	AR	Cockroach
I 25	F	27	NBA	Aspergillus fumigatus, Cockroach

BA, bronchial asthma; AR, allergic rhinitis; NBA, nasobronchial allergy; U, urticaria; ND, not determined

Supplementary Table S2. IgE reactivity profile of Austrian mugwort and ragweed allergic patients sera used for the IgE inhibition ELISA

Patient no.	Age	IgE (ELISA)				
		Art v 1	Art v 3	Art v 4	Art v 5	Amb a 1
M-R 1	18	+	+	++	+	++
M-R 2	15	-	-	-	+	++
M-R 3	22	++	++	-	++	+
M-R 4	61	++	++	++	+	++
M-R 5	39	++	++	++	-	++
M-R 6	16	++	-	++	-	+

-, no IgE reactivity; +, positive IgE reactivity; ++, positive with very high IgE reactivity

Supplementary Table S4: Allergenic protein families identified in feverfew pollen. Protein blast with sequences from Allergome database (uniprot release 29.03.2016) and constraints using the IUIS allergen nomenclature sub-committee was performed.

**Common identified proteins by (LC-MS/MS and 2-DE-LC-MS/MS)**						
**Allergenic protein family**	**Method**	**Spot #**	**Accession number**	**Description/species**	**-10lp (Score)**	**U**
60S acidic ribosomal protein	LC-MS/MS		gi|729348440	PREDICTED: 60S acidic ribosomal protein P2-4-like/*Tarenaya hassleriana*	83.88	1
	2-DE-LC-MS/MS	27	gi|802562610	PREDICTED: 60S acidic ribosomal protein P2A-like/*Jatropha curcas*	80.5	3
Aldehyde dehydrogenase	LC-MS/MS		gi|8163730	Aldehyde dehydrogenase/*Oryza sativa*	48.54	1
	2-DE-LC-MS/MS	38	gi|238846406	Aldehyde dehydrogenase 1/*Artemisia annua*	98.2	3
Aspartic peptidase (Peptidase A1 family)	LC-MS/MS		gi|976909721	Aspartic peptidase/*Cynara cardunculus*	65.02	1
	2-DE-LC-MS/MS	10	gi|976909721	Aspartic peptidase/*Cynara cardunculus*	128.7	4
Bet v 1 family	LC-MS/MS		gi|976909362	Bet v I domain-containing protein/*Cynara cardunculus*	43.38	1
	2-DE-LC-MS/MS	3	gi|167472849	Pollen allergen Que a 1 isoform/*Quercus alba*	140.04	7
Beta-1.3-glucanase (Glycosyl hydrolase 17 family)	LC-MS/MS		gi|976496731	Glycoside hydrolase/*Cynara cardunculus*	79.66	1
	2-DE-LC-MS/MS	30	gi|976928968	Glycoside hydrolase/*Cynara cardunculus*	117.38	4
Calreticulin	LC-MS/MS		gi|976913141	Calreticulin/*Cynara cardunculus*	122.41	2
	2-DE-LC-MS/MS	9	gi|976913141	Calreticulin/*Cynara cardunculus*	172.47	3
Cupin family (Germin-like protein family)	LC-MS/MS		gi|976899679	Cupin 1/*Cynara cardunculus*	38.87	1
	2-DE-LC-MS/MS	18	gi|976927409	Cupin 1/*Cynara cardunculus*	127.11	4
Cu-Zn superoxide dismutase	LC-MS/MS		gi|976902802	Superoxide dismutase. copper/zinc/*Cynara cardunculus*	199.31	5
	2-DE-LC-MS/MS	48	gi|3914999	Full=Superoxide dismutase [Cu-Zn]/*Olidago canadensis*	128.58	5
Cyclophilin	LC-MS/MS		gi|488726160	Cyclophilin/*Arachis hypogaea*	109.82	1
	2-DE-LC-MS/MS	2	gi|332806715	Cyclophilin 2/*Tagetes patula*	140.75	3
Cysteine protease (Peptidase C1 family)	LC-MS/MS		gi|1706276	Full=Cysteine proteinase inhibitor A/*Helianthus annuus*	102.47	5
	2-DE-LC-MS/MS	11	gi|28804503	Thiol protease/*Tripolium pannonicum*	120.05	3
Cytochrome c family	LC-MS/MS		gi|118012	Full=Cytochrome c/*Pastinaca sativa*	82.44	2
	2-DE-LC-MS/MS	17	gi|976899814	Cytochrome c. class I/*Cynara cardunculus*	105.41	3
Defensin-like protein	LC-MS/MS		gi|817033923	Par h I precursor/*Parthenium hysterophorus*	116.16	6
	2-DE-LC-MS/MS	1	gi|817033923	Par h I precursor/*Parthenium hysterophorus*	274.43	17
Enolase	LC-MS/MS		gi|3023685	Full=Enolase/*Alnus glutinosa*	112.7	1
	2-DE-LC-MS/MS	30	gi|976924044	Enolase/*Cynara cardunculus*	235.99	5
Glyceraldehyde-3-phosphate dehydrogenase	LC-MS/MS		gi|752855788	glyceraldehyde-3-phosphate dehydrogenase/*Lilium davidii*	130.69	1
	2-DE-LC-MS/MS	33	gi|902550458	glyceraldehyde-3-phosphate dehydrogenase/*Dendrobium catenatum*	93.05	4
Heat shock protein 70	LC-MS/MS		gi|698946330	putative heat shock protein/Taraxacum brevicorniculatum	168.93	3

	2-DE-LC-MS/MS	9	gi|976917633	Chaperone DnaK/*Cynara cardunculus*	183.9	4
LDH/MDH superfamily	LC-MS/MS		gi|976916739	Lactate dehydrogenase/*Cynara cardunculus*	138.16	1
	2-DE-LC-MS/MS	32	gi|976905497	Lactate dehydrogenase/ *Cynara cardunculus*	232.66	3
NAC-alpha family	LC-MS/MS		gi|729420144	PREDICTED: nascent polypeptide-associated complex subunit alpha-like protein 3/*Tarenaya hassleriana*	86.88	2
	2-DE-LC-MS/MS	6	gi|976923178	Nascent polypeptide-associated complex NAC domain-containing protein/*Cynara cardunculus*	184.89	4
Pathogenesis-related protein PR-1	LC-MS/MS		gi|743838314	PREDICTED: STS14 protein-like/*Populus euphratica*	62.22	1
	2-DE-LC-MS/MS	51	gi|976917157	Allergen V5/Tpx-1-related protein/*Cynara cardunculus*	89.28	4
Pectate lyase	LC-MS/MS		gi|113476	Full=Pectate lyase 1/*Ambrosia artemisiifolia*	125.1	3
	2-DE-LC-MS/MS	39	gi|302127818	putative pectate lyase precursor/*Ambrosia artemisiifolia*	148.27	4
Thaumatin-like protein	LC-MS/MS		gi|20385169	Pathogenesis-related protein 5-1/*Helianthus annuus*	57.39	1
	2-DE-LC-MS/MS	1	gi|20385169	Pathogenesis-related protein 5-1/*Helianthus annuus*	162.17	4
Thioredoxin	LC-MS/MS		gi|720041636	PREDICTED: protein disulfide-isomerase/*Nelumbo nucifera*	97.88	4
	2-DE-LC-MS/MS	36	gi|976899454	Disulfide isomerase/*Cynara cardunculus*	125.14	4
Triosephosphate-isomerase	LC-MS/MS		gi|976918965	Aldolase-type TIM barrel/*Cynara cardunculus*	103.27	3
	2-DE-LC-MS/MS	1	gi|976923382	Aldolase-type TIM barrel/*Cynara cardunculus*	265.4	10
Tubulin family	LC-MS/MS		gi|803378277	Beta-tubulin/*Camelina sp. CameTUB5*	65.03	3
	2-DE-LC-MS/MS	49	gi|902550499	Beta-1 tubulin/*Dendrobium catenatum*	167.41	3
**Proteins identified by single method (LC-MS/MS or 2-DE-LC-MS/MS)**						
Chitinase	LC-MS/MS	-	gi|425886502	Class II chitinase/*Acacia koa*	71.38	1
Chlorophyll a-b binding protein	LC-MS/MS	-	gi|146403796	chloroplast light-harvesting chlorophyll a/b-binding protein/*Artemisia annua*	50.25	1
Cobalamin-independent methionine synthase	LC-MS/MS	-	gi|976902600	Cobalamin-independent methionine synthase. partial/*Cynara cardunculus*	178.11	3
Flavodoxin	LC-MS/MS	-	gi|720077294	PREDICTED: minor allergen Alt a 7 isoform X1/*Nelumbo nucifera*	40.76	1
glutathione S-transferase	LC-MS/MS	-	gi|698502214	PREDICTED: glutathione S-transferase DHAR3/*Nicotiana sylvestris*	71.98	1
GMC oxidoreductase	LC-MS/MS	-	gi|659069842	PREDICTED: (R)-mandelonitrile lyase 1-like/*Cucumis melo*	66.88	2
Heat shock protein 90	LC-MS/MS	-	gi|702435110	PREDICTED: endoplasmin homolog /*Eucalyptus grandis*	61.82	1
Iron/manganese superoxide dismutase	LC-MS/MS	-	gi|15551753	Mn-super oxide dismutase II/*Lactuca sativa*	38.8	1
Isoflavone reductase	LC-MS/MS	-	gi|923534270	PREDICTED: isoflavone reductase homolog P3-like/*Brassica napus*	31.29	1
L3 Ribosomal protein	LC-MS/MS	-	gi|901818290	Putative 60S ribosomal protein L3/*Zostera marina*	46.21	1
Lipid transfer protein	LC-MS/MS	-	gi|118490068	Lipid transfer protein isoform 1.1 precursor/*Lactuca sativa*	74.06	1
Pectin methylesterase	LC-MS/MS	-	gi|976571059	Pectinesterase /*Cynara cardunculus*	52.26	2
Peroxiredoxin	LC-MS/MS	-	gi|823226051	PREDICTED: peroxiredoxin-2B-like/*Gossypium raimondii*	82.22	3
Polcalcin	LC-MS/MS	-	gi|976896497	Calcium-binding EF-hand/*Cynara cardunculus*	154.96	3
Polygalacturonase (Glycosyl hydrolase 28 family)	LC-MS/MS	-	gi|976926788	Glycoside hydrolase. family 28. partial/*Cynara cardunculus*	37.34	1

Profilin	LC-MS/MS	-	gi|62249502	Profilin isoallergen 1/*Ambrosia artemisiifolia*	112.17	2
Protein kinase	LC-MS/MS	-	gi|976914952	Concanavalin A-like lectin/glucanase /*Cynara cardunculus*	81.29	1
Pyrophosphatase	LC-MS/MS	-	gi|901817400	Inorganic diphosphatase/*Zostera marina*	53.11	1
Serine protease (Peptidase S8 family)	LC-MS/MS	-	gi|976923421	Peptidase S8/S53 domain-containing protein/*Cynara cardunculus*	44.97	1
Beta-xylosidase	2-DE-LC-MS/MS	30	gi|85813770	Xylan 1.4-beta-xylosidase/*Populus tremula*	88.47	3
Endochitinase (Hevein like-domain)	2-DE-LC-MS/MS	9	gi|848864792	PREDICTED: endochitinase PR4/*Erythranthe guttata*	138.39	3
Serine carboxypeptidase (Peptidase S10 family)	2-DE-LC-MS/MS	11	gi|976807531	Peptidase S10. serine carboxypeptidase/*Cynara cardunculus*	146.69	4
Xyloglucan endotransglucosylase (Glycosyl hydrolases family 16)	2-DE-LC-MS/MS	9	gi|82394883	Xyloglucan endotransglucosylase/*Gerbera hybrid cultivar*	124.43	3
Alcohol dehydrogenase	2-DE-LC-MS/MS	39	gi|970021885	PREDICTED: alcohol dehydrogenase 1/*Solanum pennellii*	93.97	4

Unprocessed image Figure 1c

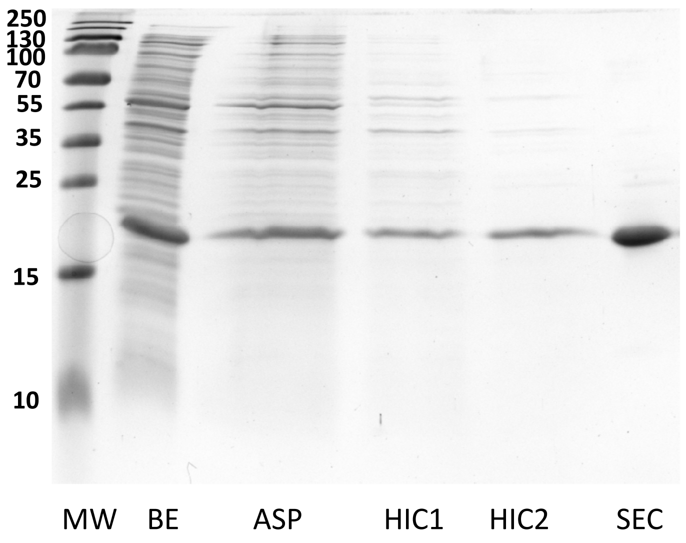

Images were taken with ChemiDoc MP Imaging System (Bio RAD) and acquired with the software Image Lab. 4.0.1. MW, molecular weight; BE, bacterial extract; ASP, ammonium sulfate precipitation; HIC1 and HIC2, hydrophobic interaction chromatography and SEC, size exclusion chromatography. The image correponding to Figure 1c was cropped (molecular weight marker) using the image tools of the same software. Processing like changing brightness and contrast were not applied.

Unprocessed images Figure 2b and Supplementary Figure S3

Figure 2b

SDS-PAGE

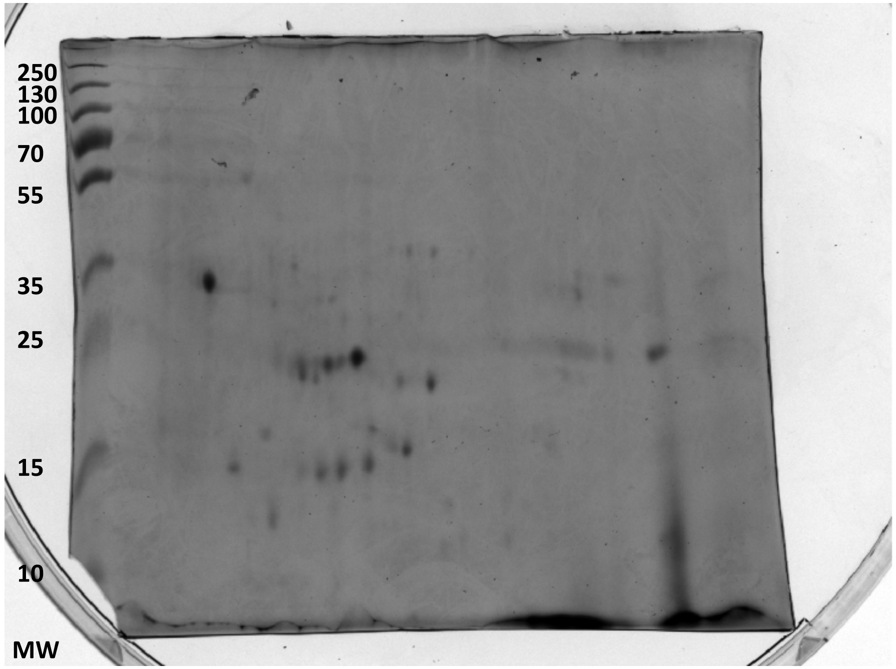

Figure S3

IMMUNOBLOT

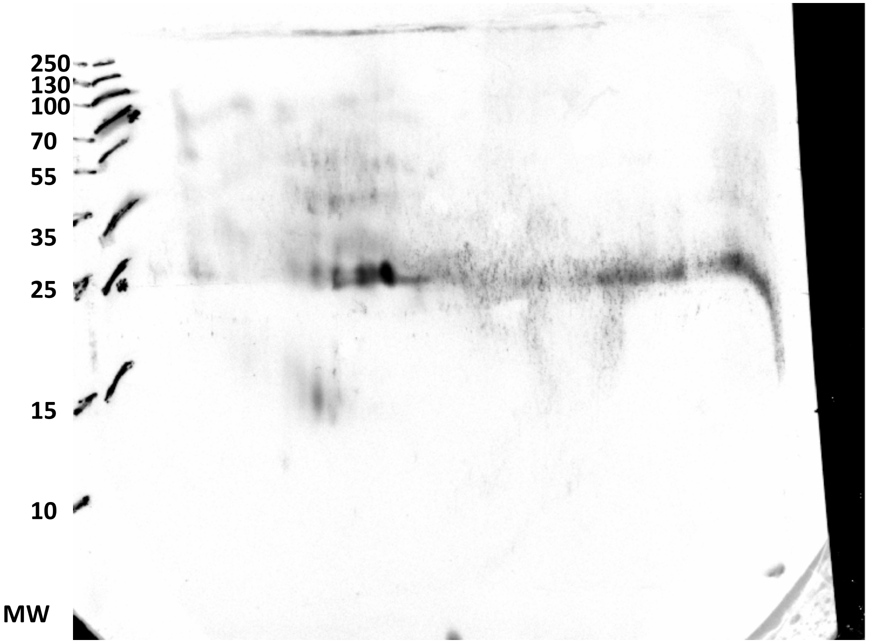

Images were taken with ChemiDoc MP Imaging System (Bio RAD) and acquired with the software Image Lab. 4.0.1. MW, molecular weight. The images correponding to Figure 2b and supplementary Figure S3 were cropped using the image tools of the same software. Processing like changing brightness and contrast were applied to the complete images.

Unprocessed image Figure 3

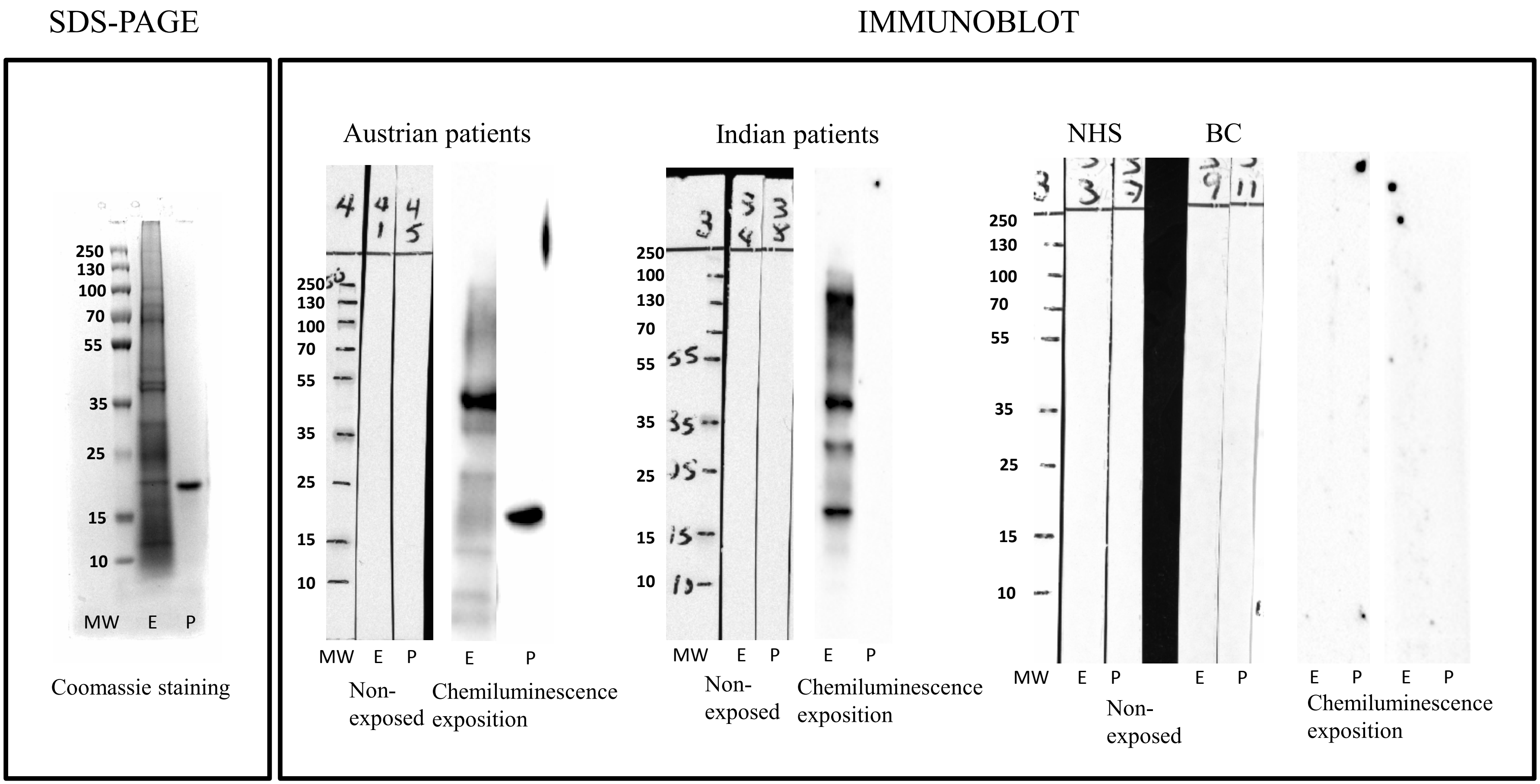

Images were taken with ChemiDoc MP Imaging System (Bio RAD) and acquired with the software Image Lab. 4.0.1. MW, molecular weight; E, pollen extract; P, recombinant Par h 1; NHS non-atopic human sera; BC, buffer control. The images correponding to Fig. 3 were cropped (in order to get straight lines) using the image tools of the same software. Processing like changing brightness and contrast were applied to all the stripes belonging to the same images.

Unprocessed image Supplementary Figure S4a and b

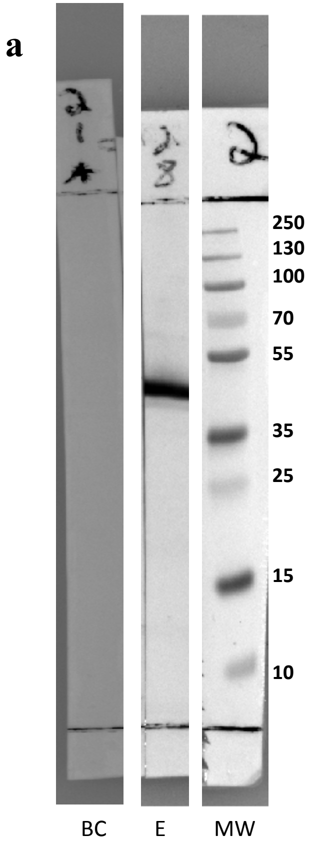

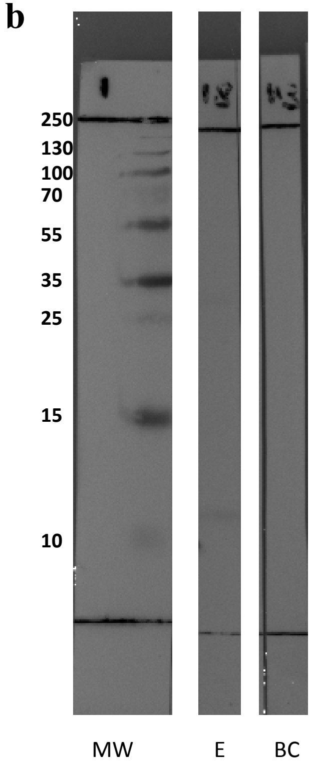

Images were taken with ChemiDoc MP Imaging System (Bio RAD) and acquired with the software Image Lab. 4.0.1. The image correponding to Supplementary Figure S4 was cropped to get straight lines, since other unrelated strips were also part of the image, using the image tools of the same software. MW, molecular weight; E, pollen extract; BC, buffer control. (**a**) The polyclonal Ab anti-Amb a 1 (stripe 2.8) and (**b**) the monoclonal Ab anti-Art v 3 (stripe 1.8) were used as primary antibodies. Processing like changing brightness and contrast were applied to all the stripes as they were part of the same images (a, b).
